# Augmented intermittent theta-burst stimulation of the left dorsolateral prefrontal cortex for cognitive dysfunction in stable-phase schizophrenia: protocol for a randomized, double-blind, sham-controlled trial

**DOI:** 10.3389/fpsyt.2025.1730074

**Published:** 2026-01-02

**Authors:** Yuke He, Shanhong Wang, Hao Wu, Wenzhong Liu, Yang Fan, Wei Xu, Han Deng, Shanshan Long, Bo Liu, Kezhi Liu, Youguo Tan

**Affiliations:** 1 The Affiliated Hospital, Southwest Medical University, Luzhou, China; 2 The Zigong Affiliated Hospital, Southwest Medical University, Zigong, China; 3 Research Center for Psychiatry, Zigong Institute of Brain Science, Zigong, China

**Keywords:** cognitive dysfunction, DLPFC, functional near-infrared spectroscopy, iTBS, rTMS, schizophrenia

## Abstract

**Background:**

Schizophrenia is a severe mental disorder affecting approximately 1% of the global population, characterized by three core symptom domains: positive symptoms, negative symptoms, and cognitive dysfunction. Repetitive transcranial magnetic stimulation (rTMS) has emerged as a promising intervention for both positive and negative symptoms in schizophrenia. However, its therapeutic potential for cognitive dysfunction remains inconclusive.

**Methods:**

This randomized, double-blind, sham-controlled trial will enroll 70 medication-stable patients meeting DSM-5 criteria for schizophrenia. Participants will be randomly allocated (1:1) to receive either active or sham augmented intermittent theta-burst stimulation (iTBS) targeting the left dorsolateral prefrontal cortex (DLPFC). The treatment protocol consists of 600 pulses per session, three sessions per day, for 10 consecutive weekdays. Clinical symptoms will be evaluated with the Positive and Negative Syndrome Scale (PANSS), cognitive function will be assessed using the Repeatable Battery for the Assessment of Neuropsychological Status (RBANS), and neural activity will be measured via functional near-infrared spectroscopy (fNIRS) during a semantic verbal fluency task. All patients will be assessed at baseline and post-intervention.

**Ethics and dissemination:**

The trial protocol complies with the principles of the Declaration of Helsinki and has been approved by the Ethics Committee of Zigong Mental Health Center (approval number: 20250802). The findings of this trial will be published and made publicly accessible in a peer-reviewed journal.

**Discussion:**

This study aims to provide strong evidence supporting the therapeutic potential of augmented iTBS for improving cognitive functioning in schizophrenia, offering a potential new treatment option for patients with schizophrenia.

**Clinical Trial Registration:**

https://www.chictr.org.cn/index.html, identifier ChiCTR2500107943.

## 1 Introduction

Schizophrenia (SZ) is a chronic, highly disabling illness. The weighted lifetime prevalence of severe psychosis is 0.6% (1), characterized by three core symptom domains: positive symptoms (e.g., hallucinations, delusions), negative symptoms (e.g., affective flattening, social withdrawal), and cognitive dysfunction (particularly in social cognition and executive functioning) (2). Among these, cognitive dysfunction plays a key role in the long-term psychosocial impairment and disability experienced by patients with schizophrenia (3). Current antipsychotic medications demonstrate limited efficacy in alleviating negative symptoms and improving cognitive functioning (4). In recent years, repetitive transcranial magnetic stimulation (rTMS) has emerged as a promising therapeutic alternative. As a noninvasive brain stimulation technique, rTMS uses pulsed magnetic fields to induce targeted electrical currents in specific cortical regions, offering potential therapeutic benefits. While rTMS has demonstrated efficacy in treating depression and received Food and Drug Administration (FDA) approval for this indication in 2008 (5), its therapeutic effectiveness for schizophrenia remains controversial (6).

The mechanisms underlying cognitive impairment in schizophrenia remain elusive, with working memory deficits representing one of its most prominent features (7). Some studies have found that structural and functional abnormalities in the dorsolateral prefrontal cortex (DLPFC) may be closely linked to cognitive impairments in patients with schizophrenia. A neuroimaging study found reduced activation of DLPFC in patients with schizophrenia while performing working memory tasks (8). Magnetic resonance imaging (MRI) data have shown that reduced cortical thickness and grey matter volume in left DLPFC (L-DLPFC) of patients with schizophrenia (9), as well as reduced grey matter volume in the prefrontal lobe, are significantly associated with working memory deficits (10). Thus, functional abnormalities in the prefrontal lobes may underlie the neurological basis of working memory deficits in schizophrenia (11, 12).

A meta-analysis has shown that rTMS of L-DLPFC is effective in improving cognitive function, but the quality of evidence from these studies is not high (13). Currently, studies attempting to alleviate cognitive symptoms by stimulating the DLPFC have not yielded consistent results. Some studies have found that high-frequency (10 Hz) stimulation of L-DLPFC can improve patients’ cognitive functions (14, 15), especially facial expression recognition and social cognition (14). However, another study has shown that the stimulation has no significant effect on cognitive function (16).

In addition to the location of stimulation, the number of stimulation pulses may also be a key factor affecting efficacy. Recent studies have found that patients with depression who were initially unresponsive to rTMS showed improvement after receiving higher doses of stimulation (17). This suggests that the standard FDA-approved protocol may be underdosed. According to the latest clinical guidelines, the FDA-approved standard intermittent theta-burst stimulation (iTBS) treatment protocol provides 600 pulses per session (5). However, a study has shown that 1800 pulses can alter cortical excitability more permanently and induce expected cellular changes more effectively (18). In addition, an augmentation protocol based on iTBS has shown significant efficacy enhancement in the treatment of depression, and this protocol can significantly increase the response and remission rates of depressed patients by intermittently administering high-frequency pulsed stimulation (19). However, this augmentation protocol has not yet been applied to patients with schizophrenia. A systematic review suggests that iTBS may be an effective means of improving cognitive function and negative symptoms in patients with schizophrenia (20); however, the optimal therapeutic regimen remains to be explored.

Based on current evidence, we propose an augmented iTBS stimulation protocol for treatment of L-DLPFC based on cognitive function-related circuits in schizophrenia. We plan to deliver 1,800 pulses—three times the standard 600-pulse burst protocol—in order to provide robust, evidence-based data for evaluating the therapeutic efficacy of high-frequency rTMS on cognitive function. The core objective of this study is to validate the safety and efficacy of our proposed augmented iTBS protocol for the treatment of schizophrenia. With this study, we hope to demonstrate that augmented iTBS protocol can have a positive impact in several key areas, including Positive and Negative Syndrome Scale (PANSS) scores on clinical symptoms, Repeatable Battery for the Assessment of Neuropsychological Status (RBANS) on cognitive functioning, and functional near-infrared spectroscopy (fNIRS) on neuroimaging, while we will monitor serious adverse events (SAE) to assess safety.

## 2 Method

### 2.1 Participant

**Figure 1:**
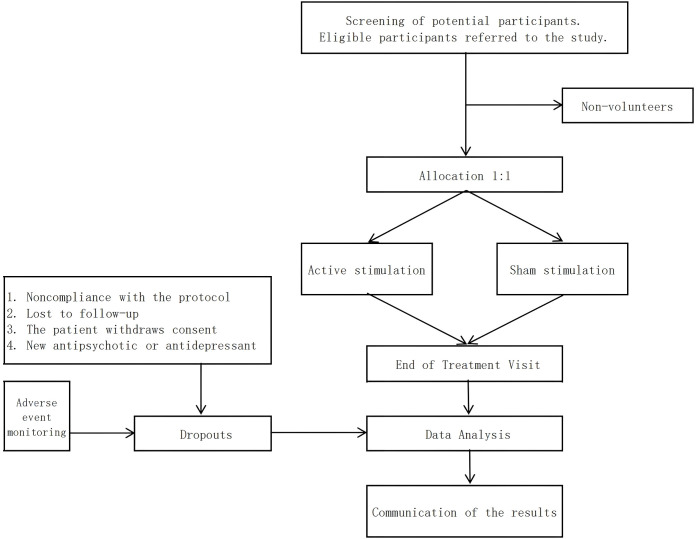
Flowchart.

We are preparing a double-blind, sham-controlled research project with two randomized subgroups aimed at investigating the clinical effectiveness of a augmented iTBS protocol for the treatment of schizophrenia. Based on a recent meta-analysis of frontal cortex rTMS for improvement of schizophrenia (21), we assumed an effect size of 0.64. Using this assumption and the baseline values as covariates, we concluded that with a power equal to or greater than 80% (β= 0.20), there was a significant difference between the two groups in terms of improvement in cognitive function (α = 0.05), a sample size of 62 calculated using G*Power, and we expected a dropout rate of 10%. Therefore, we plan to include 70 patients who met the DSM-V diagnostic criteria for schizophrenia. We will assign patients to the active stimulation and sham stimulation groups in a 1:1 ratio, and patients participating in the study will be given sequentially numbered patient codes to which the corresponding treatment modality (active or sham stimulation) will be assigned (**Figure 1**). The subjects for this study will be recruited from the inpatients of Zigong Mental Health Centre, who need to meet all the inclusion criteria and not meet any exclusion criteria.

**Figure 2:**
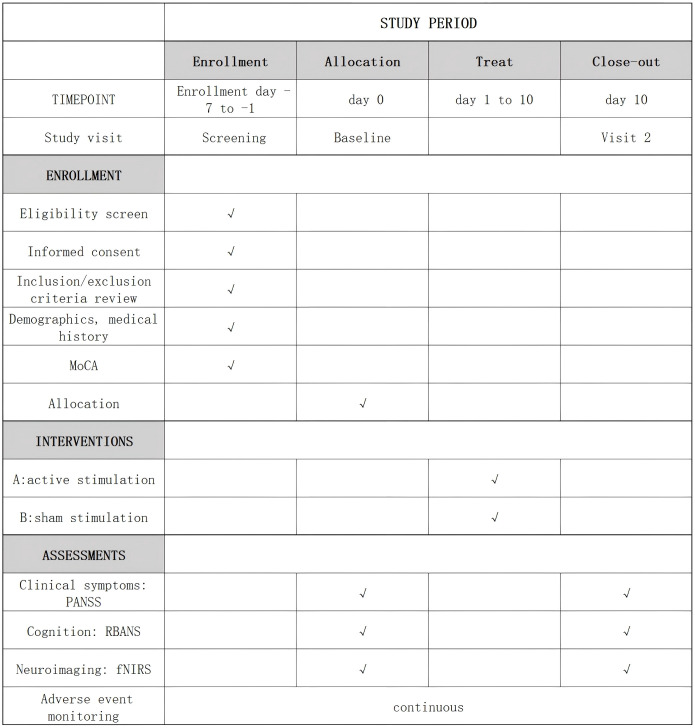
Enrollment schedule, treatment, and outcome measures. MoCA, Montreal Cognitive Assessment; PANSS, Positive and Negative Syndrome Scale; RBANS, Repeatable Battery for the Assessment of Neuropsychological Status; fNIRs, functional near-infrared spectroscopy.

Clinical and cognitive assessments, along with fNIRS measurements, will be conducted by an independent rater blinded to the treatment condition at two time points: baseline and post-intervention (day 10)(**Figure 2**).

### 2.2 Inclusion criteria

1) Meeting the diagnostic criteria for schizophrenia according to the Diagnostic and Statistical Manual of Mental Disorders, Fifth Edition; 2) Stable clinical status and taking antipsychotic medication: continuously taking a stable dose of antipsychotic medication for more than 4 weeks; 3) Age between 18 and 60 years; 4) Montreal Cognitive Assessment(MoCA) score of 19-25 (22), able to cooperate in completing the scale; 5) Right-handed.

### 2.3 Exclusion criteria

1) Presence of any significant neurological disorder; 2) Presence of mental retardation; 3) History of seizures or presence of epileptic activity on baseline electroencephalogram (EEG) (subject to evaluation by clinical EEG and epileptologist); 4) Alcohol or drug abuse within the last 3 months; 5) Depressive episodes or antidepressant treatment within the last 4 weeks; 6) History of electroconvulsive therapy (ECT); 7) Have a pacemaker, drug pump, cochlear implant, defibrillator, neurostimulator, or other metal device implanted in the body that is incompatible with rTMS; 8) Presence of severe damage to the skin surface in the area of cranial stimulation; 9) Being pregnant; 10) Presence of a serious physical illness; 11) Presence of severe positive symptoms that may interfere with the administration of cognitive tests.

### 2.4 Randomization

To ensure the scientific rigor and balance of sample allocation, this study will employ a stratified randomization method to evenly distribute 70 samples into two groups. The aim is to minimize the bias in sample allocation and enhance the reliability and reproducibility of the experimental results. Gender is an important potential confounding factor that may influence the study outcomes. Therefore, the samples will be stratified by gender into two layers: the male stratum and the female stratum. Within each stratum, a random number generator will be used to assign a random number to each sample. The random numbers will be generated following a uniform distribution ranging from 0 to 1. Subsequently, the samples within each stratum will be sorted based on the magnitude of the random numbers. The first 50% of samples within each stratum will be allocated to the active stimulation group, while the remaining 50% will be allocated to the sham stimulation group.

### 2.5 Blinding

Patients and raters will not be informed about treatment allocation, and the researcher providing the treatment will not be involved in any assessment. All efficacy assessments will be conducted by blinded researchers who will not be involved in the treatment process. Patients will be advised not to disclose any details of the treatment process to the raters. To test the effectiveness of blinding, at the end of treatment visit (visit 2), we will ask the raters and the subjects to guess the group allocation and analyze their guesses to verify whether the blinding was successful.

### 2.6 Intervention

rTMS will be performed using CYY-IIIB Magnetic Stimulator (Yiruide Company, Ltd, Wuhan, China). Subjects in the active stimulation group will receive 10 consecutive days of iTBS mode three times per day, with at least 1 h of interval between each intervention. The stimulation site for the L-DLPFC will be determined using the 10–20 EEG localization system, with stimulation intensity set at 80% of the resting motor threshold (RMT).

**Figure 3:**
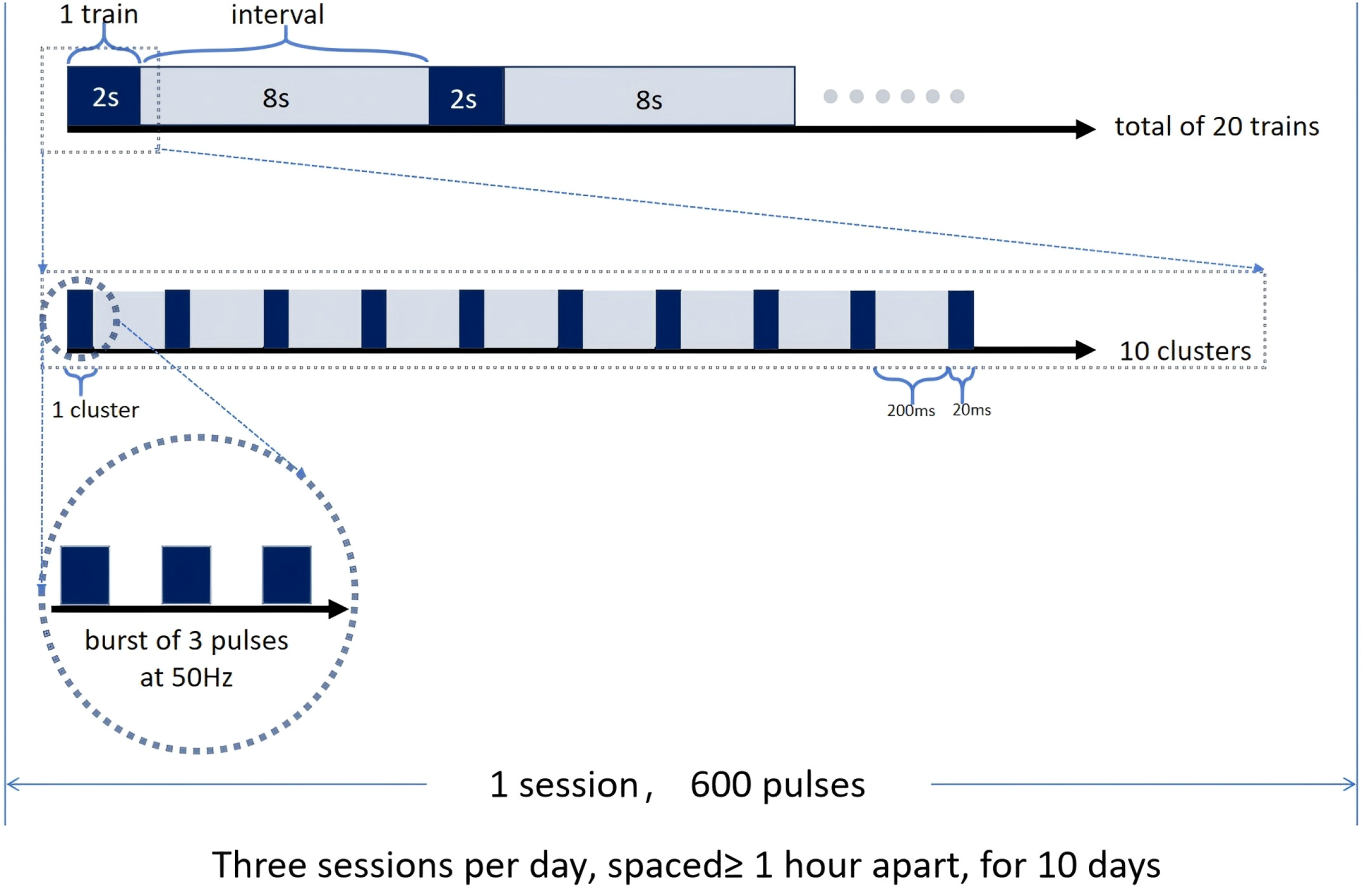
iTBS intervention mode, Each session consists of 20 trains, each lasting 2 seconds with an 8-second inter-train interval. Within each train, 10 clusters are delivered at 5 Hz, each cluster containing 3 pulses at 50Hz. Each cluster lasts 20ms, and the interval between two clusters is 200ms. Three sessions will be conducted each day, with at least a 1-hour interval between sessions, for a total of 10 days.

Each session will consist of 20 trains, each lasting 2 seconds with an 8-second inter-train interval. Within each train, 10 clusters will be delivered, each cluster containing 3 pulses. The intra-cluster stimulation frequency will be 50 Hz, and the inter-cluster frequency was 5 Hz. The coil will be flipped 90° to deliver sham stimulation. The sham stimulation will also generate a magnetic field that can be felt by the participant, but the field does not penetrate the skull. We plan to deliver 1800 pulses per day to the L-DLPFC in three sessions (with a minimum of 1 h between stimulations) for a total of 10 min per day for 10 days (**Figure 3**).

### 2.7 Outcome

#### 2.7.1 Primary outcome

Our primary outcome will be the mean change scores of cognitive function assessed using RBANS from baseline to the end of the treatment period. The RBANS, developed by Randolph in 1998, assesses five cognitive domains through 12 subtests, including Attention (Digit Breadth and Encoding Tests), Verbal (Picture Naming and Semantic Fluency Tests), Visual Breadth (Picture Copying and Line Orientation Tests), Immediate Memory (Vocabulary Acquisition and Story Repetition Tests), and Delayed Memory (Vocabulary Recall, Vocabulary Re-recognition, Story Recall, and Picture Recall Tests). We expect that patients receiving active stimulation will show significant improvements in performance on these tests, scoring higher on the total score of the RBANS than the sham stimulation group.

#### 2.7.2 Secondary outcomes

The secondary outcome measures of our study will include the changes in the average score of the negative scale of the PANSS scale, the average change in oxyhemoglobin (oxyHb) and deoxyhemoglobin (deoxyHb) in the cerebral cortex.

We will use PANSS to assess the improvement of patients’ clinical symptoms. PANSS is a standardized tool for assessing the severity of symptoms in patients with schizophrenia. It was first proposed by Kay et al. in 1987 (23) and has been widely used in clinical and research settings and has become the gold standard for assessing psychotic symptoms through semi-structured interviews (24). It contains three factors: positive symptoms, negative symptoms, and general psychopathology, with a total of 30 items, each of which is scored on a 7-point scale from 1 (asymptomatic) to 7 (very severe). The total score ranges from 30 to 210, with higher scores indicating more severe symptoms. Given that cognitive impairment is strongly correlated with negative symptoms in patients with schizophrenia (21, 25), we hypothesize that the decrease in PANSS negative symptom scores will be significantly greater in patients receiving active stimulation than in those receiving sham stimulation.

**Figure 4:**
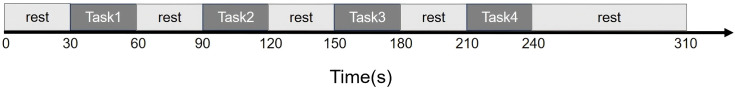
fNIRS procedure. The fNIRS testing utilizes a semantic VFT, which comprises four tasks, each preceded by a 30-second rest period, with a 70-second rest period following the final task.

We will use fNIRS technology to record the changes in hemoglobin concentration in the prefrontal cortex during the semantic Verbal Fluency Task (VFT). The VFT is a neurocognitive task commonly used in fNIRS studies and can be divided into phonological and semantic VFTs. The semantic VFT in this experiment will be divided into four phases, and each phase will consist of a 30s resting period and a 30s task period, with a 70s resting period at the end, for a total of 310s (**Figure 4**). During the resting period, the participants will be asked to repeatedly count ‘1, 2, 3, 4, 5’, and during the task period, they will be asked to say as many words as possible belonging to the categories of ‘four-legged animals…’, ‘fruits…’, ‘household appliances’, and ‘vegetables’, respectively, and the researcher recorded the number of words that were correct without repetition. The researcher will record the number of correct words without repetitions. Before starting the task, the participants will be given a practice session to ensure that they understood the task. A meta-analysis has shown that patients with multiple psychiatric disorders experience hypoactivation (e.g., decreased ΔHbO) in relevant brain regions during the VFT task, which may be associated with impaired cognitive function (26). Therefore, we plan to compare fNIRS responses between the two groups, and we expect greater activation in relevant brain regions in the active stimulation group.

#### 2.7.3 Safety monitoring

We will closely monitor possible serious adverse events (SAEs) during treatment. Although previous studies have shown that iTBS treatment is safe and well tolerated in the treatment of depression (27) and schizophrenia (28), based on previous experience, the most common side effect is mild headache, and the most serious side effect is seizure, which is very rare (incidence is approximately 1 in 10,000). Our hypothesis is that there will be no significant difference in the incidence of SAEs between the two groups of patients. If SAEs occur, patients will stop treatment immediately and be unblinded.

### 2.8 Statistical analysis of data

The normality of the measures will be assessed using the Shapiro-Wilk test. The Levene test will be used to assess the conformity of the variance of the measures. Two independent samples t-test will be used for measures that met normality. The Mann-Whitney U test will be used for measures that did not meet normality. Categorical variables will be analyzed using the chi-square test. Pearson’s correlation analysis will be used to explore the relationship between cognitive function improvement and changes in neural activity. P-value <0.05 will indicate a statistically significant difference.

### 2.9 Analysis sets

We will use the following analysis sets:

1. The intention-to-treat (ITT) analysis set will include all participants who were randomly assigned to a treatment group, irrespective of treatment adherence or study completion. This approach aims to assess the treatment effect under real-world conditions by providing a conservative estimate of efficacy that accounts for non-adherence and dropout, thereby reducing bias and reflecting the treatment’s potential effectiveness in clinical practice.
2. Safety analysis set: This will consist of a subset of participants in the randomized analysis set who will have received at least one pulse stimulation treatment with at least one safety evaluation.
3. Per-protocol analysis set: This will consist of a subset of subjects in the full analysis set who will be at least 80% compliant (having received 80% of the planned stimuli) and will not have any program bias, which is considered to have a substantial impact on the primary efficacy outcome.

### 2.10 Withdrawal of patient from study

Patients may withdraw from the study at any time for any reason without affecting their future medical care by the investigator or study site. Every effort will be made to keep the patient in the study by documenting the reasons why the patient did not complete the treatment and/or did not complete the study. Patients may withdraw from the study for any of the following reasons:

1. Noncompliance with the protocol or serious breach of the protocol.
2. Severe or intolerable adverse event (AE).
3. Failure to follow up.
4. Withdrawal of consent by the patient.
5. Adjustment of the medication regimen or receipt of Modified Electroconvulsive Therapy (MECT) during the study.

When a patient withdraws from active participation in the study, the investigator will document the reason for discontinuation on the relevant page of the Case Report Form. If possible, all patients who discontinue treatment or withdraw from the study early will be assessed at the time of the early withdrawal visit. The investigator should make diligent efforts and explore all possible options to contact patients who fail to attend the visits. It is crucial to obtain follow-up data from any patient who withdraws from the study due to an AE. If a patient must withdraw from the study because of a SAE, the patient will be followed up until the condition is stable or the event is no longer considered clinically significant. In each case, efforts must be made to conduct the safety and follow-up procedures as specified in the protocol.

## 3 Discussion

This study is a double-blind, sham-controlled, parallel-group trial designed to investigate the effects of augmented iTBS on the L-DLPFC and its associated cerebral cortical oxyhemoglobin levels, in patients with schizophrenia using fNIRS techniques. Given the causal relationship between cognitive deficits and impaired daily functioning in patients with schizophrenia and the potential of rTMS as a cognitive enhancement treatment, an in-depth understanding of the mechanisms of action of rTMS in schizophrenia is essential for optimizing interventions (6). The exact mechanism by which rTMS positively affects cognition is not yet known. Neuropsychological evidence suggests that the frontal lobes play a key role in social cognition, and that rTMS targeting the L-DLPFC in patients with schizophrenia may enhance cognitive processing (14). Clarifying the relationship between prefrontal modulation and cognitive improvement will provide a framework for interventions, as prefrontal cognitive deficits are the root of much of the disability in schizophrenia. The results of this study could help optimize rTMS protocols for the treatment of schizophrenia and provide insight into the neurocognitive mechanisms underlying the interventions.

fNIRS is a noninvasive neuroimaging technique that indirectly reflects neural activity by measuring changes in oxyHb and deoxyHb in the cerebral cortex. Increased neuronal activity in a brain region leads to elevated local blood flow, typically characterized by higher oxyHb and lower deoxyHb levels (29), which fNIRS can detect to infer brain activation. fNIRS-derived measures of cerebral oxygenation (e.g., changes in oxyHb and deoxyHb) hold promise as potential biomarkers for schizophrenia. Several studies have found significant differences in activation patterns or hemodynamic parameters in specific brain regions in patients with schizophrenia (30, 31), and these differences may help in the early diagnosis of schizophrenia or in distinguishing between different subtypes of patients. Furthermore, this technique has been widely applied to monitor cognitive function during rehabilitation in patients with stroke and brain injury (18).

In addition, this study will provide preliminary safety and efficacy data on augmented iTBS treatment protocol to support future larger clinical trials. Positive results may drive more extensive testing of this promising tool to alleviate the severe symptoms affecting most people with schizophrenia. In conclusion, this study will integrate multiple approaches to optimize noninvasive brain stimulation for cognitive enhancement in schizophrenia, highlighting its critical importance as a therapeutic strategy for this disorder.

## 4 Trail status

The protocol version number is V1.0, and the date is July 23, 2025. The Ethics Committee of Zigong Mental Health Center approved the study protocol on August 2,2025(approval number 20250802). The trial was registered on August 21,2025(ChiCTR2500107943). The trial started on September 1, 2025. The trial is currently recruiting participants. We predict that the recruitment will be completed by January 31, 2026.

## References

[1] Huang Y Wang Y Wang H Liu Z Yu X Yan J . Prevalence of mental disorders in China: a cross-sectional epidemiological study. Lancet Psychiatry. (2019) 6:211–24. doi: 10.1016/S2215-0366(18)30511-X, PMID: 30792114

[2] Jauhar S Johnstone M McKenna PJ . Schizophrenia. Lancet. (2022) 399:473–86. doi: 10.1016/S0140-6736(21)01730-X, PMID: 35093231

[3] Balogh N Egerházi A Berecz R Csukly G . Investigating the state-like and trait-like characters of social cognition in schizophrenia: a short term follow-up study. Schizophr Res. (2014) 159:499–505. doi: 10.1016/j.schres.2014.08.027, PMID: 25305062

[4] Fusar-Poli P Papanastasiou E Stahl D Rocchetti M Carpenter W Shergill S . Treatments of negative symptoms in schizophrenia: meta-analysis of 168 randomized placebo-controlled trials. Schizophr Bull. (2015) 41:892–9. doi: 10.1093/schbul/sbu170, PMID: 25528757 PMC4466178

[5] Chen L Fukuda AM Jiang S Leuchter MK van Rooij SJH Widge AS . Treating depression with repetitive transcranial magnetic stimulation: A clinician’s guide. Am J Psychiatry. (2025) 182:525–41. doi: 10.1176/appi.ajp.20240859, PMID: 40302403 PMC12323729

[6] Sciortino D Pigoni A Delvecchio G Maggioni E Schiena G Brambilla P . Role of rTMS in the treatment of cognitive impairments in Bipolar Disorder and Schizophrenia: a review of Randomized Controlled Trials. J Affect Disord. (2021) 280:148–55. doi: 10.1016/j.jad.2020.11.001, PMID: 33212406

[7] Lett TA Voineskos AN Kennedy JL Levine B Daskalakis ZJ . Treating working memory deficits in schizophrenia: a review of the neurobiology. Biol Psychiatry. (2014) 75:361–70. doi: 10.1016/j.biopsych.2013.07.026, PMID: 24011822

[8] Semkovska M Bédard MA Stip E . Hypofrontality and negative symptoms in schizophrenia: synthesis of anatomic and neuropsychological knowledge and ecological perspectives. Encephale. (2001) 27:405–15., PMID: 11760690

[9] Wheeler AL Chakravarty MM Lerch JP Pipitone J Daskalakis ZJ Rajji TK . Disrupted prefrontal interhemispheric structural coupling in schizophrenia related to working memory performance. Schizophr Bull. (2014) 40:914–24. doi: 10.1093/schbul/sbt100, PMID: 23873858 PMC4059434

[10] Goghari VM Macdonald AW 3rd Sponheim SR . Relationship between prefrontal gray matter volumes and working memory performance in schizophrenia: a family study. Schizophr Res. (2014) 153:113–21. doi: 10.1016/j.schres.2014.01.032, PMID: 24529364 PMC4144341

[11] Quidé Y Morris RW Shepherd AM Rowland JE Green MJ . Task-related fronto-striatal functional connectivity during working memory performance in schizophrenia. Schizophr Res. (2013) 150:468–75. doi: 10.1016/j.schres.2013.08.009, PMID: 24016726

[12] Faget-Agius C Boyer L Lançon C Richieri R Fassio E Soulier E . Structural and functional reorganization of working memory system during the first decade in schizophrenia. A cross-sectional study. Schizophr Res. (2013) 151:48–60. doi: 10.1016/j.schres.2013.10.023, PMID: 24230490

[13] Du XD Li Z Yuan N Yin M Zhao XL Lv XL . Delayed improvements in visual memory task performance among chronic schizophrenia patients after high-frequency repetitive transcranial magnetic stimulation. World J Psychiatry. (2022) 12:1169–82. doi: 10.5498/wjp.v12.i9.1169, PMID: 36186505 PMC9521529

[14] Wölwer W Lowe A Brinkmeyer J Streit M Habakuck M Agelink MW . Repetitive transcranial magnetic stimulation (rTMS) improves facial affect recognition in schizophrenia. Brain Stimul. (2014) 7:559–63. doi: 10.1016/j.brs.2014.04.011, PMID: 24857264

[15] Chen X Ji GJ Zhu C Bai X Wang L He K . Neural correlates of auditory verbal hallucinations in schizophrenia and the therapeutic response to theta-burst transcranial magnetic stimulation. Schizophr Bull. (2019) 45:474–83. doi: 10.1093/schbul/sby054, PMID: 29733409 PMC6403092

[16] Wobrock T Guse B Cordes J Wölwer W Winterer G Gaebel W . Left prefrontal high-frequency repetitive transcranial magnetic stimulation for the treatment of schizophrenia with predominant negative symptoms: a sham-controlled, randomized multicenter trial. Biol Psychiatry. (2015) 77:979–88. doi: 10.1016/j.biopsych.2014.10.009, PMID: 25582269

[17] Hadley D Anderson BS Borckardt JJ Arana A Li X Nahas Z . Safety, tolerability, and effectiveness of high doses of adjunctive daily left prefrontal repetitive transcranial magnetic stimulation for treatment-resistant depression in a clinical setting. J ECT. (2011) 27:18–25. doi: 10.1097/YCT.0b013e3181ce1a8c, PMID: 21343710

[18] Volz LJ Benali A Mix A Neubacher U Funke K . Dose-dependence of changes in cortical protein expression induced with repeated transcranial magnetic theta-burst stimulation in the rat. Brain Stimul. (2013) 6:598–606. doi: 10.1016/j.brs.2013.01.008, PMID: 23433874

[19] Cole EJ Phillips AL Bentzley BS Stimpson KH Nejad R Barmak F . Stanford neuromodulation therapy (SNT): A double-blind randomized controlled trial. Am J Psychiatry. (2022) 179:132–41. doi: 10.1176/appi.ajp.2021.20101429, PMID: 34711062

[20] Kishi T Ikuta T Sakuma K Hamanaka S Nishii Y Hatano M . Theta burst stimulation protocols for schizophrenia: A systematic review and network meta-analysis. JAMA Netw Open. (2024) 7:e2441159. doi: 10.1001/jamanetworkopen.2024.41159, PMID: 39446321 PMC11581676

[21] Aleman A Enriquez-Geppert S Knegtering H Dlabac-de Lange JJ . Moderate effects of noninvasive brain stimulation of the frontal cortex for improving negative symptoms in schizophrenia: Meta-analysis of controlled trials. Neurosci Biobehav Rev. (2018) 89:111–8. doi: 10.1016/j.neubiorev.2018.02.009, PMID: 29471017

[22] Wu C Dagg P Molgat C . A pilot study to measure cognitive impairment in patients with severe schizophrenia with the Montreal Cognitive Assessment (MoCA). Schizophr Res. (2014) 158:151–5. doi: 10.1016/j.schres.2014.07.006, PMID: 25092174

[23] Kay SR Fiszbein A Opler LA . The positive and negative syndrome scale (PANSS) for schizophrenia. Schizophr Bull. (1987) 13:261–76. doi: 10.1093/schbul/13.2.261, PMID: 3616518

[24] Liechti S Capodilupo G Opler DJ Opler M Yang LH . A developmental history of the positive and negative syndrome scale (PANSS). Innov Clin Neurosci. (2017) 14:12–7., PMID: 29410932 PMC5788246

[25] Tseng PT Zeng BS Hung CM Liang CS Stubbs B Carvalho AF . Assessment of noninvasive brain stimulation interventions for negative symptoms of schizophrenia: A systematic review and network meta-analysis. JAMA Psychiatry. (2022) 79:770–9. doi: 10.1001/jamapsychiatry.2022.1513, PMID: 35731533 PMC9218931

[26] Yeung MK Lin J depression P . schizophrenia, and other psychiatric disorders using fNIRS and the verbal fluency test: A systematic review and meta-analysis. J Psychiatr Res. (2021) 140:416–35. doi: 10.1016/j.jpsychires.2021.06.015, PMID: 34146793

[27] Blumberger DM Vila-Rodriguez F Thorpe KE Feffer K Noda Y Giacobbe P . Effectiveness of theta burst versus high-frequency repetitive transcranial magnetic stimulation in patients with depression (THREE-D): a randomised non-inferiority trial. Lancet. (2018) 391:1683–92. doi: 10.1016/S0140-6736(18)30295-2, PMID: 29726344

[28] Chauhan P Garg S Tikka SK Khattri S . Efficacy of intensive cerebellar intermittent theta burst stimulation (iCiTBS) in treatment-resistant schizophrenia: a randomized placebo-controlled study. Cerebellum. (2021) 20:116–23. doi: 10.1007/s12311-020-01193-9, PMID: 32964381 PMC7508243

[29] Kim H Choi J Jeong B Fava M Mischoulon D Park MJ . Impaired oxygenation of the prefrontal cortex during verbal fluency task in young adults with major depressive disorder and suicidality: A functional near-infrared spectroscopy study. Front Psychiatry. (2022) 13:915425. doi: 10.3389/fpsyt.2022.915425, PMID: 35815016 PMC9260011

[30] Zhu J Zhuo C Xu L Liu F Qin W Yu C . Altered coupling between resting-state cerebral blood flow and functional connectivity in schizophrenia. Schizophr Bull. (2017) 43:1363–74. doi: 10.1093/schbul/sbx051, PMID: 28521048 PMC5737873

[31] Rubinov M Bullmore E . Schizophrenia and abnormal brain network hubs. Dialogues Clin Neurosci. (2013) 15:339–49. doi: 10.31887/DCNS.2013.15.3/mrubinov, PMID: 24174905 PMC3811105

